# Love and Truth: What Really Matters for Children Born Through Third-Party Assisted Reproduction

**DOI:** 10.1111/cdep.12406

**Published:** 2021-06

**Authors:** Susan Golombok

**Affiliations:** Centre for Family Research, University of Cambridge

**Keywords:** assisted reproduction, donor conception, surrogacy

## Abstract

Ever since the birth of the first baby born through in vitro fertilization in 1978, advances in reproductive technologies have raised new concerns about the outcomes for children. In this article, I summarize research on children born through assisted reproduction involving a third party, that is, children born through egg donation, sperm donation, and surrogacy, with particular attention to the findings of a longitudinal study of children born to heterosexual couples in the United Kingdom. The assisted reproduction families generally showed high levels of family functioning and children’s adjustment from early childhood through to adolescence, suggesting that biological relatedness is less important than positive parent–child relationships for the well-being of children conceived by third-party assisted reproduction. Similarly, studies of families created by third-party reproduction with two mothers, single mothers, two fathers, and single fathers have shown that these families function well.

In 1978, Louise Brown was the first baby born through in vitro fertilization (IVF). Ever since the birth of Brown—the first child conceived in a laboratory—advances in reproductive technologies have raised new concerns about the outcomes for children: Are donor-conceived children harmed by the absence of a genetic link to a parent or by the secrecy about their genetic origins? Are children born through surrogacy distressed by the knowledge that the surrogate gave them away to their intended parents? Are children conceived by sperm donation to single mothers or lesbian couples adversely affected by not knowing the identity of their biological father? And are children born through surrogacy to gay fathers at increased risk of developing psychological problems due to the absence of a mother? In this article, I summarize research on children born through assisted reproduction involving a third party, that is, children born through egg donation, sperm donation, and surrogacy. These children, who lack a gestational or genetic connection to one or both parents, face challenges that children born though IVF alone do not. In the article, I integrate findings from the six phases of the U.K. Longitudinal Study of Assisted Reproduction Families from a developmental perspective. (For a broader discussion of the research and information on the experiences of the families, see Golombok, 2020a, 2020b; Imrie & Golombok, 2020.)

Two key concerns have been raised regarding donor-conceived children. The first is that nongenetic parents—mothers in families formed by egg donation and fathers in families formed by sperm donation—may be distant from or hostile toward their children. This idea comes from clinical studies on difficulties experienced by fathers of children born through sperm donation (Baran & Pannor, 1993) and from evolutionary psychology, which draws on research on families with stepfa-thers to suggest lower quality relationships between nongenetic parents and their children (Daly & Wilson, 1989). The second concern, from studies of adoptive families (Brodzinsky, 2011) and from theory and research on family therapy (Papp, 1993), is that secrecy about children’s genetic origins can harm children’s psychological well-being. Additional misgivings have been expressed regarding children born through surrogacy, including the assumption that knowing that the woman who gave birth to them had relinquished them to other parents would cause them psychological harm, the potentially negative impact of the involvement of a surrogate on the relationship between the mother and the child, and stigmatization surrounding this controversial route to parenthood.

The U.K. Longitudinal Study of Assisted Reproduction Families, a longitudinal study of parent-child relationships and children’s adjustment, sought to examine these issues in 51 egg donation families, 50 sperm donation families, 42 surrogacy families, and a comparison group of 80 families formed through unassisted conception; families were studied when their children were 1, 2, 3, 7, 10, and 14 years old. These children were born at the millennium, and the parents were predominantly White, well educated, and financially stable, which was typical of families formed through assisted reproduction at that time. In the following sections, I summarize the study’s findings in early childhood, middle childhood, and adolescence and discuss associated developmental challenges. The term *assisted reproduction families* refers to the egg donation, sperm donation, and surrogacy families combined, and the term *donor conception families* refers to the egg and sperm donation families combined.

## Early Childhood

The transition to parenthood, which can be stressful for all parents, presents specific challenges for mothers and fathers of children born through third-party assisted reproduction. These include the experience of infertility, intrusive and often prolonged fertility treatment, and the need to come to terms with the involvement of a gamete donor or surrogate, all of which may adversely affect the relationship between parents and their children. From the perspective of attachment theory, the social context of the family is an important influence on parents’ representations of their relationship with their child (George & Solomon, 1999; Slade, Belsky, Aber, & Phelps, 1999) because social and psychological challenges may interfere with parents’ thoughts and feelings about their child and consequently, their parenting behavior and children’s adjustment.

Contrary to predictions that these families would experience difficulties, according to the study, mothers in families formed by donor conception had higher levels of warmth, enjoyment of parenthood, and emotional involvement with their 1-year-olds than mothers of infants born through unassisted conception (Golombok, Lycett, et al., 2004), as did mothers of infants born through surrogacy (Golombok, Murray, Jadva, MacCallum, & Lycett, 2004). When the children were 1 year old, mothers who had conceived through egg donation did not differ from mothers who had conceived through sperm donation.

When their children were 2 years old, as assessed by the Parent Development Interview (Slade et al., 1999), mothers of children born through donor conception took greater pleasure in their toddlers than mothers of children born through unassisted conception, and mothers of children conceived by egg donation were similar to the sperm donation mothers on this variable (Golombok, Jadva, Lycett, Murray, & MacCallum, 2005). Moreover, the egg and sperm donation mothers had similarly low levels of anger, guilt, and disappointment in their children as the unassisted conception mothers, and each other. The only difference identified between mothers of children born through egg donation and mothers of children born through sperm donation was less overprotection by the egg donation mothers. Similarly, the mothers of 2-year-old children born through surrogacy showed higher levels of pleasure in their children than the mothers of children born through unassisted conception. They also showed lower levels of anger, guilt, and disappointment in their children than the mothers whose children were born through unassisted conception (Golombok, MacCallum, Murray, Lycett, & Jadva, 2006). When the children were 3 years old, the mothers of children born through assisted reproduction showed a higher quality of parenting than the mothers whose children were born through unassisted conception (Golombok, Murray, et al., 2006). Parenting quality did not differ between the surrogacy mothers and the egg donation mothers, or between the egg donation mothers and the sperm donation mothers.

In terms of the quality of their relationships with their 1- and 2-year-olds, fathers of children born by sperm donation were similar to fathers of children born using donated eggs or through unassisted conception (Golombok, Lycett, et al., 2004; Golombok et al., 2005), apart from lower levels of emotional involvement with their 1-year-olds by fathers of children born through sperm donation. Fathers of children born through surrogacy showed more positive parenting than fathers of children born through unassisted conception when their children were 1 year old (Golombok, Murray, et al., 2004), and less stress associated with parenting when their children were 2 (Golombok, MacCallum, et al., 2006). (Data were not collected from fathers when the children were 3 years old.) Consistent with the findings on parent–child relationships, the children were functioning well, with no differences in levels of psychological problems in the egg donation, sperm donation, and surrogacy families when the children were 2 years old (Golombok, MacCallum, et al., 2006; Golombok et al., 2005) and 3 years old (Golombok, Murray, et al., 2006).

Therefore, in the early years, the absence of a gestational or genetic connection between parents and their children did not appear to interfere with the development of positive relationships between them. Instead, almost all the differences identified in the study indicated more positive outcomes for the assisted reproduction families than the unassisted conception families, probably reflecting the commitment to and pleasure in their children by parents who had struggled to start a family. These findings are consistent with those of a qualitative study that found that, although some egg donation mothers took time to feel that the infant was their own, they felt secure and confident in their position as their child’s mother by the end of the first year (Imrie, Jadva, & Golombok, 2020).

## Middle Childhood

Preschool children have, at most, a rudimentary grasp of reproduction. However, children’s transition to school corresponds with their increased social understanding (Hughes, 2011) and specifically, with their greater comprehension of the concepts of family, inheritance, and genetic relatedness (Richards, 2000; Solomon, Johnson, Zaitchik, & Carey, 1996; Williams & Smith, 2010), with most children acquiring a biological concept of family at around age 7 (Solomon et al., 1996). This is also when children who have been adopted develop more understanding of what it means to be adopted, including the loss of their birth parents (Brodzinsky, 2011). Thus, children conceived using donated eggs or sperm, or born through surrogacy, who are aware of the nature of their conception are likely to develop a greater understanding of their origins at this time. One of the most hotly debated issues in the field of assisted reproduction is whether parents should be open with their children about their origins (Nuffield Council on Bioethics, 2013). Parents of children born through third-party assisted reproduction are faced with the difficult questions of whether, when, and what to disclose to their children about their birth.

The donor conception families generally showed a high quality of parenting in middle childhood. However, by the time their children were 7, only 41% of the egg donation parents and 28% of the sperm donation parents had told their children about their genetic origins (Readings, Blake, Casey, Jadva, & Golombok, 2011). Mother–child relationships in the families that did not disclose this information were less positive in terms of warmth, sensitivity, and quality of interaction than in the unassisted conception families and also in terms of quality of interaction compared to the families that disclosed the information (Golombok, Readings, Blake, Casey, Mellish, et al., 2011). Although parents who do not tell their children about their donor conception may be less communicative generally, not just about the child’s conception, these findings suggest that openness with children about their origins before they start school is important for donor conception parents, just as it is for adoptive parents. Disclosing families did not differ from nondisclosing families with respect to negative aspects of parenting, such as conflict and hostility.

Fathers of children born via sperm donation were similar to egg donation fathers and fathers of children born through unassisted conception regarding the quality of their relationships with their 7-year-olds, showing equally high levels of warmth, involvement, control, discipline, and interaction. However, children who were conceived via sperm donation showed greater negativity toward their fathers in an interaction task than children born through egg donation or unassisted conception (Casey, Jadva, Readings, Blake, & Golombok, 2013). Although comparisons of father–child relationships were not conducted between disclosing and nondisclosing families, the greater negativity shown by children conceived by sperm donation may be associated with the high levels of nondisclosure in these families. The children themselves continued to show high levels of adjustment at 7 and 10 years (Golombok, Blake, Casey, Roman, & Jadva, 2013).

In contrast to the donor conception parents, almost all the surrogacy parents in the study had been open with their children about the circumstances of their birth before the children reached school age; the absence of a pregnancy makes it very difficult to conceal surrogacy. When the children were 7 years old, the families created through surrogacy were similar to the families formed though unassisted conception with respect to the quality of relationships between mothers and their children, apart from less positive mother–child interaction (Golombok, Readings, Blake, Casey, Marks, et al., 2011). However, the surrogacy children exhibited higher levels of psychological difficulties at age 7 than the egg or sperm donation children, who were similar to each other and to the children born through unassisted conception (Golombok et al., 2013). The higher levels of psychological problems in surrogacy children at age 7 are consistent with the increase in adjustment difficulties among internationally adopted children at this age (Stams, Juffer, Rispens, & Hoksbergen, 2000); for the adopted children, this was linked to looking different than their parents and having to deal with identity issues at a younger age than children who are not adopted (Juffer & van IJzendoorn, 2005). Children born through surrogacy, who were usually aware of the circumstances of their birth and the identity of their surrogate, may experience the same challenges. The less positive mother–child interaction in surrogacy families when children were 7 may be associated with the children’s increased levels of adjustment problems at this age. By age 10, the surrogacy children no longer differed from the other children in terms of psychological adjustment, a finding that is consistent with internationally adopted children when they were assessed at adolescence (Juffer & van IJzendoorn, 2005).

## Adolescence

For children born through assisted reproduction, as for adopted children, adolescence brings specific issues, especially regarding identity formation. Donor-conceived adolescents who are aware of their origins are faced with the challenge of incorporating an understanding of themselves as having a genetic connection to a donor and possibly donor siblings—genetic half-siblings born from the same donor who are growing up in different families—whose identities they may never know. Children born through surrogacy face similar challenges in relation to their gestational connection to the surrogate and their genetic connection to her if they were conceived using surrogate’s egg, as well as a gestational or genetic link to the surrogate’s own children. Adolescence is also associated with more conflict with parents, particularly with respect to the desire for greater autonomy (Smetana, Campione-Barr, & Metzger, 2006; Smetana & Rote, 2019), which may present greater difficulties in assisted reproduction families where parents tend to be very involved with their children.

The sixth phase of the U.K. Longitudinal Study of Assisted Reproduction Families was conducted when the children reached 14, the age when children have acquired an understanding of degrees of biological relatedness (Richards, 2000; Williams & Smith, 2010). The families continued to function well. Mothers in surrogacy families showed greater acceptance of their children, less negative parenting, and more positive family functioning than mothers of children born through donor conception. To the extent that the increased levels of adjustment difficulties shown by the surrogacy children at age 7 were associated with identity issues, surrogacy parents’ openness with their children in their early years may have enabled these children to develop a secure sense of identity during middle childhood, resulting in better relationships with their mothers by adolescence. Although it is often assumed that adolescents would be distressed about their birth through surrogacy, when interviewed about their thoughts, feelings, and experiences about surrogacy, most were unconcerned, with only one adolescent expressing some unhappiness, and a few saw it as an advantage (Zadeh, Ilioi, Jadva, & Golombok, 2018).

In the donor conception families, the egg donation families showed poorer outcomes than the sperm donation families, in terms of both family functioning and mothers’ acceptance of their adolescent children (Golombok, Ilioi, Blake, Roman, & Jadva, 2017). These differences were apparent from information obtained independently from both mothers and adolescents, which provides greater confidence in the findings. Thus, it appears that the lack of a genetic connection between mothers and children in egg donation families poses a challenge to the mother–child relationship at adolescence. Mothers who are genetically unrelated to their children may find their adolescents’ interest in their origins and their desire for autonomy problematic. Adolescence may present even greater difficulties for egg donation mothers who have not informed their children that they were conceived by a donor, although this issue was not studied directly.

In the study, adolescents in all family types showed high levels of adjustment, self-esteem, and psychological well-being. Although disclosing, nondisclosing, and unassisted conception families did not differ overall, adolescents who knew about their origins since preschool showed more positive family relationships and psychological well-being at age 14 than adolescents who did not know that early (Ilioi, Blake, Jadva, Roman, & Golombok, 2017), again indicating that early disclosure to children is associated with more positive psychological outcomes. This is likely because they have had the opportunity to gradually assimilate information about their origins according to their level of social understanding.

## Conclusions

Despite concerns to the contrary, in the U.K. Longitudinal Study of Assisted Reproduction Families, the assisted reproduction families generally showed high levels of family functioning and children’s adjustment from early childhood through to adolescence. The differences identified did not point to dysfunctional family relationships but instead reflected variation within the expected range. The idea that third-party assisted reproduction adversely affects parenting and children’s adjustment comes, in part, from research showing an increased likelihood of childhood psychological problems in adoptive families (Palacios & Brodzinsky, 2010) and stepfamilies (Dunn, Deater-Deckard, Pickering, O’Connor, & Golding, 1998), in which children similarly lack a biological link to one or both parents. However, the problems experienced by adopted children and stepchildren often arise from difficult family situations before being adopted, or before or after moving into a stepfamily. Adopted children often have suffered maltreatment before being placed with their adoptive parents, sometimes for years, and many have been moved from one foster family to another before being adopted (Palacios & Brodzinsky, 2010). Children in stepfamilies often have been separated from a parent to whom they were attached and are required to form relationships with new family members. Moreover, stepparents generally do not see stepchildren as their own children (Dunn, Davies, O’Connor, & Sturgess, 2000). In contrast, children born through assisted reproduction are raised from birth by parents who wanted to have them and who consider them to be their own children. Biological relatedness seems to be less important for the well-being of children conceived by third-party assisted reproduction than are warm and responsive relationships between parents and their children.

Although the absence of a biological connection between children and their parents does not appear to cause difficulties for children, not telling children about their origins or delaying disclosure beyond the preschool years is associated with less positive outcomes for adolescents’ well-being and family relationships. Moreover, just because adolescents born through donor conception and surrogacy are functioning well does not mean that their donor or surrogate is of no significance to them. Some donor-conceived adolescents have little interest in finding out about their donor. But others search for information on the Internet. In investigations of motivations, adolescents and young adults who searched for their sperm donor and donor siblings were curious about resemblances in physical and personality characteristics, wanted to learn about their ancestry, and wished for a more complete story of how they were born (Canzi, Accordini, & Facchin, 2019; Jadva, Freeman, Kramer, & Golombok, 2010; Scheib, McCormick, Benward, & Ruby, 2020; Scheib, Ruby, & Benward, 2017). In many cases, these youth were more interested in their donor siblings than in their donors; they wanted information about their donor, and some wanted to meet him, but they usually did not see him as their father, and they were more likely to develop enduring connections with their donor siblings. In a study that explored why some adolescents are more interested in their donor relations than others, secure attachment relationships with mothers were associated with greater acceptance of and curiosity about donor conception (Slutsky et al., 2016).

The U.K. Longitudinal Study of Assisted Reproduction Families collected data from mothers, fathers, children, and teachers using standardized interviews, observational assessments, and standardized questionnaires. Nevertheless, it is the only prospective, comparative study of parent–child relationships and children’s psychological adjustment in families formed through sperm donation, egg donation, surrogacy, and unassisted conception and the only in-depth study of children born through surrogacy. Therefore, replication should be a priority, especially given the increasing numbers of children being born through assisted reproduction worldwide. Because the parents in the study were predominantly White and of medium to high socioeconomic status, the findings may not be relevant to more diverse families, who may struggle financially to afford fertility treatment, or who are from religious or ethnic backgrounds that do not accept third-party assisted reproduction, requiring parents to keep their children’s biological origins secret.

The study also focused on children born to heterosexual couples. More research is being done on lesbian-mother families formed by sperm donation (Bos & Gartrell, 2020), single hetero-sexual-mother families formed by sperm donation (Golombok, Zadeh, Freeman, Lysons, & Foley, 2020; Golombok, Zadeh, Imrie, Smith, & Freeman, 2016), families with gay fathers created through surrogacy and egg donation (Golombok, Blake, et al., 2017; Rubio et al., 2020), and single-father families with children born through surrogacy and egg donation (Carone, Baiocco, Lingiardi, & Barone, 2020), all with similarly positive outcomes. These findings show that families created by third-party reproduction with two mothers, single mothers, two fathers, or single fathers function well, irrespective of the number, gender, and sexual orientation of the parents. As with traditional families formed by assisted reproduction, in nontraditional families, the quality of parent–child relationships appears to be more important for children than the way in which the family is constructed.

The overall findings of research on children born through third-party assisted reproduction are consistent with a relational developmental systems framework (Osher, Cantor, Berg, Steyer, & Rose, 2020; Overton, 2015): Relationships, such as those between parents and children, and context, such as the disclosure or nondisclosure of children’s biological origins, interact reciprocally with characteristics of the child to influence development. Newly emerging family forms raise new questions about the psychological consequences for children. Transgender parents who have had children through fertility preservation and lesbian couples that use one partner’s egg to create the other partner’s pregnancy are just two examples of 21st-century families made possible through advances in assisted reproduction. Researchers need to study the outcomes of parents and children in these families. However, based on what we know from current studies, warm and supportive relationships between parents and their children, and openness about the children’s origins, seem to be what matter most for children born through third-party assisted reproduction.
